# Low-threshold amplified spontaneous emission and lasing from colloidal nanocrystals of caesium lead halide perovskites

**DOI:** 10.1038/ncomms9056

**Published:** 2015-08-20

**Authors:** Sergii Yakunin, Loredana Protesescu, Franziska Krieg, Maryna I. Bodnarchuk, Georgian Nedelcu, Markus Humer, Gabriele De Luca, Manfred Fiebig, Wolfgang Heiss, Maksym V. Kovalenko

**Affiliations:** 1 Department of Chemistry and Applied Biosciences, Laboratory of Inorganic Chemistry, ETH Zürich, Vladimir-Prelog-Weg 1, CH-8093 Zürich, Switzerland; 2 Laboratory for Thin Films and Photovoltaics, Empa—Swiss Federal Laboratories for Materials Science and Technology, Überlandstrasse 129, CH-8600 Dübendorf, Switzerland; 3 Institute of Semiconductor and Solid State Physics, University Linz, Altenbergerstraße 69, 4040 Linz, Austria; 4 Department of Materials, Laboratory for Multifunctional Ferroic Materials, ETH Zürich, Vladimir-Prelog-Weg 4, CH-8093 Zürich, Switzerland; 5 Materials for Electronics and Energy Technology (i-MEET), Friedrich-Alexander-Universität Erlangen-Nürnberg, Martensstraße 7, 91058 Erlangen, Germany; 6 Energie Campus Nürnberg (EnCN), Fürther Straße 250, 90429 Nürnberg, Germany

## Abstract

Metal halide semiconductors with perovskite crystal structures have recently emerged as highly promising optoelectronic materials. Despite the recent surge of reports on microcrystalline, thin-film and bulk single-crystalline metal halides, very little is known about the photophysics of metal halides in the form of uniform, size-tunable nanocrystals. Here we report low-threshold amplified spontaneous emission and lasing from ∼10 nm monodisperse colloidal nanocrystals of caesium lead halide perovskites CsPbX_3_ (X=Cl, Br or I, or mixed Cl/Br and Br/I systems). We find that room-temperature optical amplification can be obtained in the entire visible spectral range (440–700 nm) with low pump thresholds down to 5±1 μJ cm^−2^ and high values of modal net gain of at least 450±30 cm^−1^. Two kinds of lasing modes are successfully observed: whispering-gallery-mode lasing using silica microspheres as high-finesse resonators, conformally coated with CsPbX_3_ nanocrystals and random lasing in films of CsPbX_3_ nanocrystals.

## Abstract (web summary)

Lead halide perovskite colloidal nanocrystals have promising optoelectronic properties, such as high photoluminescence quantum yields and narrow emission linewidths. Here, the authors report low-threshold amplified spontaneous emission and two kinds of lasing in nanostructured caesium lead halide perovskites.

## Graphical abstract

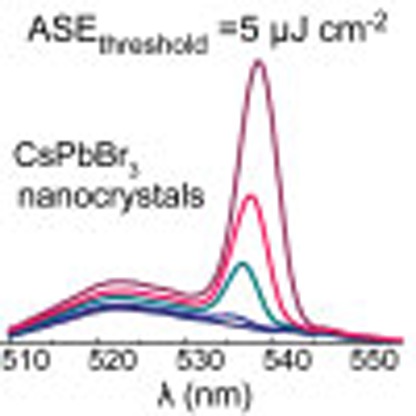

Recent years have seen multiple reports demonstrating outstanding optoelectronic characteristics of metal halide semiconductors with perovskite crystal structures, in the form of thin films, microcrystals and bulk single crystals^1^,^2^,^3^,^4^,^5^,^6^,^7^,^8^,^9^,^10^,^11^. In particular, hybrid organic–inorganic lead halide perovskites such as MAPbX_3_ (where MA=methyl ammonium and X=Cl, Br or I, or mixed Cl/Br and Br/I systems) have shown great potential as both light-absorbing and light-emitting direct-bandgap solution-deposited semiconductors. As absorber layers, MAPbX_3_ materials have enabled inexpensive solar cells with certified power conversion efficiencies of up to 20% (NREL efficiency chart, www.nrel.gov)^12^ and highly sensitive solution-cast photodetectors operating in the visible^13^, ultraviolet^14^ and X-ray^15^ spectra regions. Owing to their bright photoluminescence (PL), MAPbX_3_ thin films and nanowires have been used in electrically driven light-emitting diodes^16^ and as optical gain media for lasing^17^,^18^,^19^,^20^,^21^. We have recently shown that similarly high optoelectronic quality is also accessible in fully inorganic CsPbX_3_ analogues, when these compounds are synthesized in the form of colloidal nanocrystals (NCs)^22^. In particular, CsPbX_3_ NCs exhibit bright emission with PL quantum yields (QYs) reaching 90% and narrow emission linewidths of 70–100 meV (12–40 nm, for PL peaks from 410 to 700 nm, correspondingly). Precise and continuous tuning of bandgap energies over the entire visible spectral region is achievable foremost via compositional control (mixed halide Cl/Br and Br/I systems), but also through quantum-size effects. CsPbX_3_ NCs appear to be largely free from mid-gap trap states, similar to their MAPbX_3_ cousins^23^. Both molecular solutions of MAPbX_3_ and colloidal solutions of CsPbX_3_ NCs share the common feature of facile solution deposition on arbitrary substrates. Further, CsPbX_3_ NCs are readily miscible with other optoelectronic materials (polymers, fullerenes and other nanomaterials) and feature surface-capping ligands for further adjustments of the electronic and optical properties, and solubility in various media. We also note that CsPbX_3_ NCs are formed in the pure cubic perovskite phase, in which PbX_6_ octahedra are three-dimensionally interconnected by corner-sharing. In contrast, their bulk counterparts exist exclusively in wider-bandgap one-dimensional orthorhombic phases at ambient conditions^24^,^25^,^26^. This disparity is most pronounced for red-emitting CsPbI_3_ NCs that exhibit a narrow gap of down to 1.75 eV, whereas the corresponding bulk material has a *ca.* 1 eV larger bandgap and is yellow-colored and non-luminescent. A key practical advantage of CsPbX_3_ NCs is the facile access to the blue–green spectral region of 410–530 nm via one-pot synthesis^22^. In comparison, common metal chalcogenide colloidal quantum dots such as CdSe NCs need to be extremely small (≤5 nm) to emit in the blue–green, and as-synthesized they exhibit rather low PL QYs of ≤5% due to mid-gap trap states. In addition, they are chemically and photochemically unstable, and require coating with an epitaxial layer of a more chemically robust, wider-gap semiconductor, such as CdS. On the other hand, narrow emission linewidths of *ca.* 100 meV, high PL QYs of up 90% and high photochemical stability have been achieved for Cd-chalcogenide NCs as the result of two decades of research efforts to precisely engineer core-shell morphologies with independent control of the core and shell compositions and thicknesses (for example, CdSe_core_/ZnCdS_shell_ or ‘giant-shell' CdSe_core_/CdS_shell_)^27^,^28^,^29^,^30^ and anisotropic CdSe–CdS dot-in-rod and platelet-like morphologies^31^,^32^. Overall, each of these two families of colloidal semiconductors—CsPbX_3_ NCs and Cd-chalcogenide nanostructures—feature their respective advantages.

Inspired by the highly efficient PL of CsPbX_3_ NCs, in this study we investigate the possibility of using CsPbX_3_ NCs as an inexpensive optical gain medium. First, for thin films of CsPbX_3_ NCs, we report the observation of amplified spontaneous emission (ASE), tunable over most of the visible range (440–700 nm) with low pump thresholds down to 5±1 μJ cm^−2^ and high values of modal net gain of at least 450 cm^−1^. Among other colloidal semiconductor materials, such low-threshold pump fluencies have only been previously demonstrated for colloidal CdSe and CdSe/CdS nanoplatelets^33^,^34^,^35^, whereas CdSe/ZnCdS NCs exhibit higher thresholds (from 800 μJ cm^−2^ in the blue to 90 μJ cm^−2^ in the red)^28^ presumably due to stronger Auger recombination^36^. We then realize two different lasing regimes for CsPbX_3_ NCs depending on the resonator configuration: whispering-gallery-mode (WGM) lasing using single silica microsphere resonators, conformally coated with CsPbX_3_ NCs, and random lasing in CsPbX_3_ NC films.

## Results

### Basic characteristics of CsPbX_3_ NCs in solutions and in films

**Figure 1:**
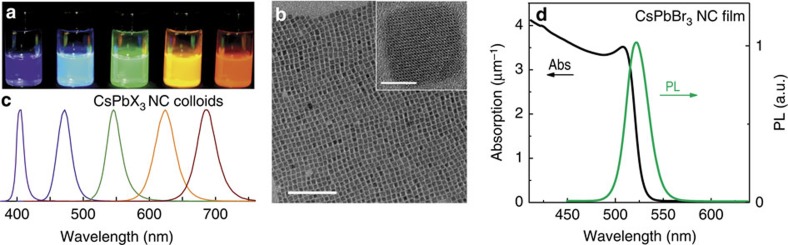
Colloidal caesium lead halide perovskite NCs. (**a**) Stable dispersions in toluene under excitation by a ultraviolet lamp (*λ*=365 nm). (**b**) Low- and high-resolution transmission electron microscopy images of CsPbBr_3_ NCs; corresponding scale bars are 100 and 5 nm. (**c**) PL spectra of the solutions shown in (**a**). (**d**) Optical absorption and PL spectra of a *ca.* 400-nm-CsPbBr_3_ NC film.

As-synthesized CsPbX_3_ NCs, capped with oleylamine and oleic acid as surface ligands, form stable colloidal dispersions in typical nonpolar solvents such as toluene (Fig. 1a)^22^. For the spectroscopic studies in this work, we selected monodisperse samples of cubic-shaped NCs with mean sizes of *ca.* 9–10 nm (Fig. 1b). These NCs readily form uniform, compact films of sub-micron thickness on drop-casting onto a glass substrate.

The bandgap of CsPbX_3_ NCs is controlled via compositional modulations, for example, by altering the Cl/Br ratio for the 410–530 nm range, and Br/I ratio for the 530–700 nm range (Fig. 1c). A pronounced excitonic peak is preserved in the absorption spectrum of the CsPbBr_3_ NC film (Fig. 1d). The absorption coefficients of the densely packed films are in the range of (3.6–4.0)·10^4^ cm^−1^ (or 3.6–4.0 μm^−1^), indicating that up to 70–80% of the pumping laser light (*λ*=400 nm) is absorbed by 300–400-nm thick films. The refractive index of a CsPbBr_3_ NC film is estimated to be 1.85–2.30 at 400–530 nm from the optical reflectance and absorption spectra (Supplementary Fig. 1). The PL from this NC film exhibits a peak with a narrow linewidth of 25 nm (110 meV), Stokes-shifted by 13 nm (57 meV) with respect to the excitonic absorption peak (Fig. 1d). The PL QYs of the same NCs in the solution reach values of up to 70–90% (for green-to-red-emitting NCs) indicating a high degree of electronic surface passivation. The PL lifetimes are very similar for solutions and for films (Supplementary Fig. 2; 1–22 ns, longer for lower-bandgap NCs). PL excitation spectrum from an NC film closely resembles the absorption spectrum (Supplementary Fig. 3).

### Amplified spontaneous emission from CsPbX_3_ NCs

**Figure 2:**
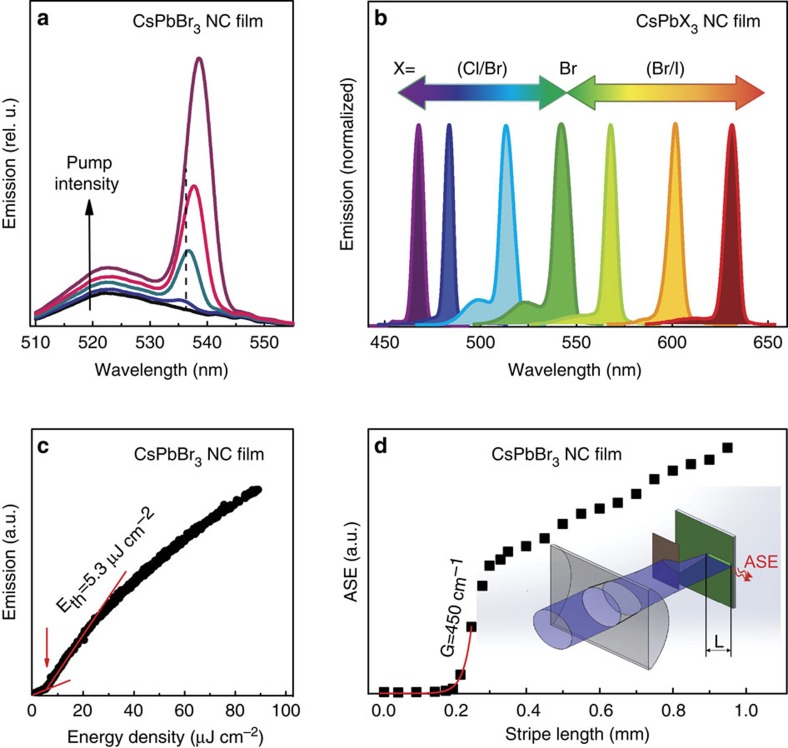
ASE spectra from thin films of CsPbX_3_ NCs. (**a**) Pump-fluence dependence of the emission from a CsPbBr_3_ NC film (pumping intensity range was 3–25 μJ cm^−2^). (**b**) Spectral tunability of ASE via compositional modulation. (**c**) Threshold behaviour for the intensity of the ASE band of the CsPbBr_3_ NC film shown in (**a**). (**d**) Variable stripe-length experiment for estimation of modal net gain for the CsPbBr_3_ NC film. All spectra were excited at *λ*=400 nm with 100 fs laser pulses.

Clear signatures of the ASE emission—narrowing of the emission peaks and threshold behaviour with a steep rise in intensity above the threshold—are readily obtained from 300- to 400-nm thick films produced by drop-casting colloidal solutions onto glass substrates (Fig. 2a; excitation at 400 nm with 100 fs pulses; and Supplementary Fig. 4 presenting ASE/PL on a logarithmic scale in a wider range of pumping intensities). ASE is spectrally different from PL emission; it has a narrower bandwidth of 4–9 nm (full width at half maximum, FWHM; see Supplementary Fig. 5) due to a narrow gain in bandwidth and is red-shifted by *ca.* 10 nm with respect to the PL maximum. When the ASE spectrum is overlaid with the Tauc plot of the direct-bandgap absorption (Supplementary Fig. 3), the ASE spectral maxima coincide with the end of the shallow absorption tail (Urbach tail). This red-shifted ASE may have its origins in re-absorbance during single-exciton lasing^28^,^29^ or in the excitonic binding energies in the bi-excitonic optical gain mechanism^34^,^37^. Similar to PL spectra, ASE can be obtained in the whole visible spectral region by varying the composition of CsPbX_3_ NCs (Fig. 2b). The threshold for building ASE is *ca.* 5±1 μJ cm^−2^ (Fig. 2c) for CsPbBr_3_ perovskite NCs, and generally falls in the range of 5–22 μJ cm^−2^ for all other compositions (Supplementary Fig. 6). Notably, the ASE linewidth increases for samples with a lower ASE threshold suggesting that a larger portion of the emission falls within the optical gain conditions, enlarging the optical gain bandwidth.

In addition to the ASE threshold, the net modal gain is an important figure-of-merit that, from a practical point of view, indicates the efficiency of light amplification in the material and the quality of the resonator needed for achieving lasing^38^. Optical gain can be measured by using the variable stripe length method^39^, where the excitation light is shaped into a line of variable lengths on the sample surface (see the schematics in the inset of Fig. 2d and a photograph of the emitted light in Supplementary Fig. 7), and the emission intensity is then measured as a function of stripe length, *L*. When the stripe length reaches the threshold value where propagation losses are compensated by the optical amplification, the PL spectrum starts to show an additional ASE component that grows with stripe length (see Fig. 2d and corresponding spectra in Supplementary Fig. 8). The threshold region can be fitted with the model of net modal gain (*G*): 
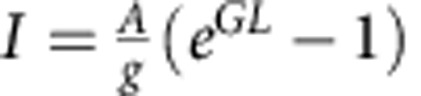
, yielding high values of *G* ranging from 450 to 500 cm^−1^. Considering these gain values, the build-up time for ASE was estimated to be 140 fs, considerably shorter than the ASE threshold lifetime of 300 ps.

**Figure 3:**
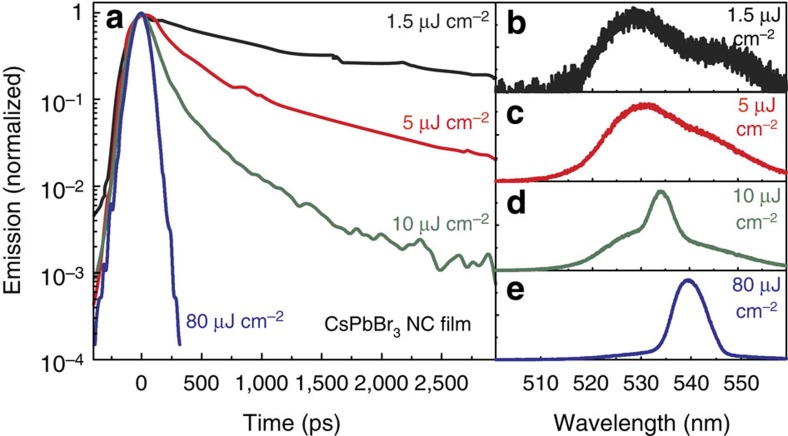
Time-resolved measurements from the CsPbBr_3_ NC film. (**a**) Decay traces at pump fluences varied from subthreshold to well above ASE threshold values with emission recorded at ASE peak wavelengths. (**b**–**e**) The corresponding full emission spectra.

**Figure 4:**
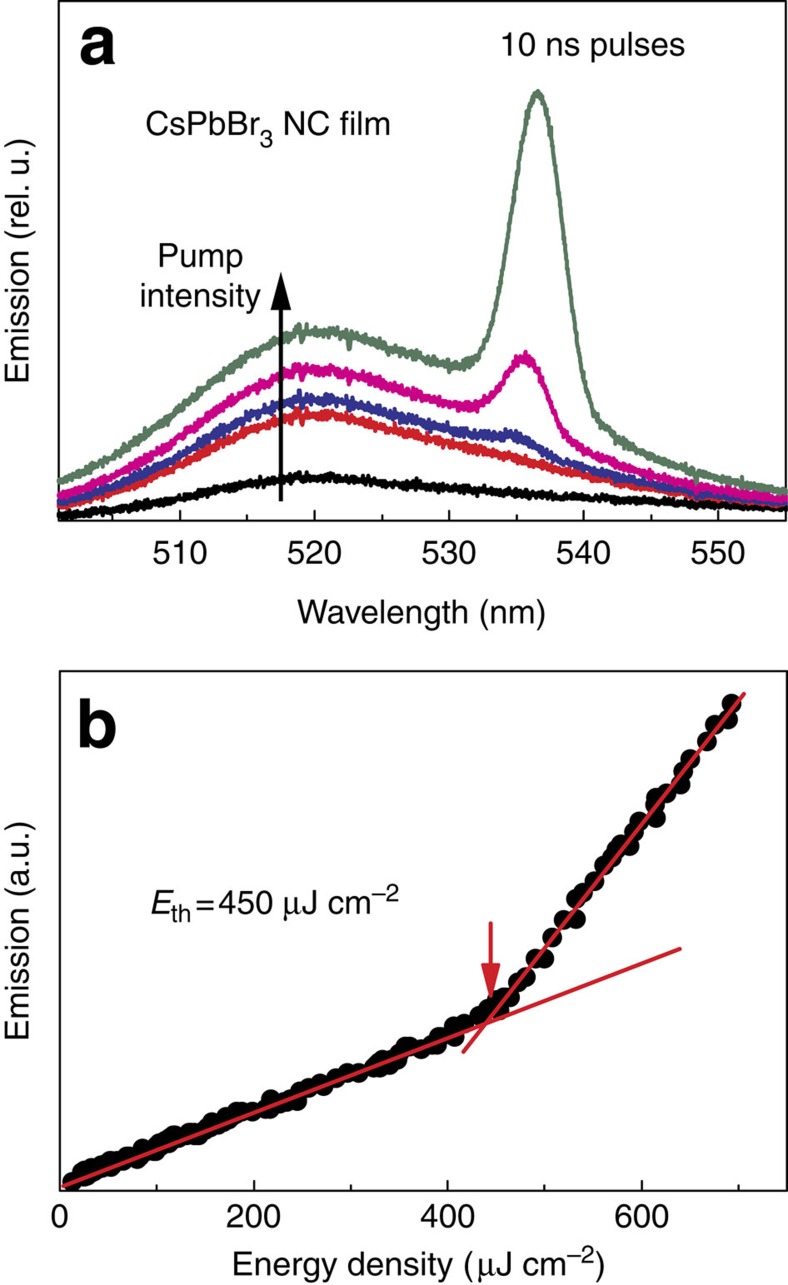
ASE from CsPbBr_3_ NC film under nanosecond excitation. (**a**) Evolution of the emission spectra with the increase of pumping fluence and (**b**) corresponding dependence of the emission at 535 nm on pump fluence. Spectra were excited at *λ*=355 nm with 10 ns laser pulses.

Another characteristic and expected feature of ASE, seen in time-resolved experiments (Fig. 3), is the acceleration of radiative recombination due to switch from individual to collective emission. For CsPbBr_3_ NC films in this work, at excitation intensities lower than the ASE threshold (<2 μJ cm^−2^; Fig. 3b), typical PL lifetimes of several nanoseconds are observed with nearly single-exponent behaviour. Well above ASE thresholds (80 μJ cm^−2^; Fig. 3e), ASE lifetimes of 60 ps were estimated, again with clean, single-exponent line shape. At the ASE threshold, an ASE lifetime of 300 ps can be roughly estimated from a bi-exponential fit assuming competing ASE and PL processes. A ‘quasi-continuous wave' regime of excitation can be observed with pumping pulses of longer duration than this ASE lifetime^27^, though at the expense of higher overall pumping fluence to maintain the same instant excitation intensity over the whole pulse duration. Such pulses can be provided by conventional, inexpensive nanosecond lasers. In this case, with a 300-ps ASE lifetime at 5–10 μJ cm^−2^ femtosecond pumping thresholds, we estimate a threshold fluence of 150–300 μJ cm^−2^ for 10-ns excitation pulses. In close agreement, ASE thresholds of 400–500 μJ cm^−2^ were observed experimentally (Fig. 4).

### Whispering-gallery-mode lasing from CsPbX_3_ NCs

**Figure 5:**
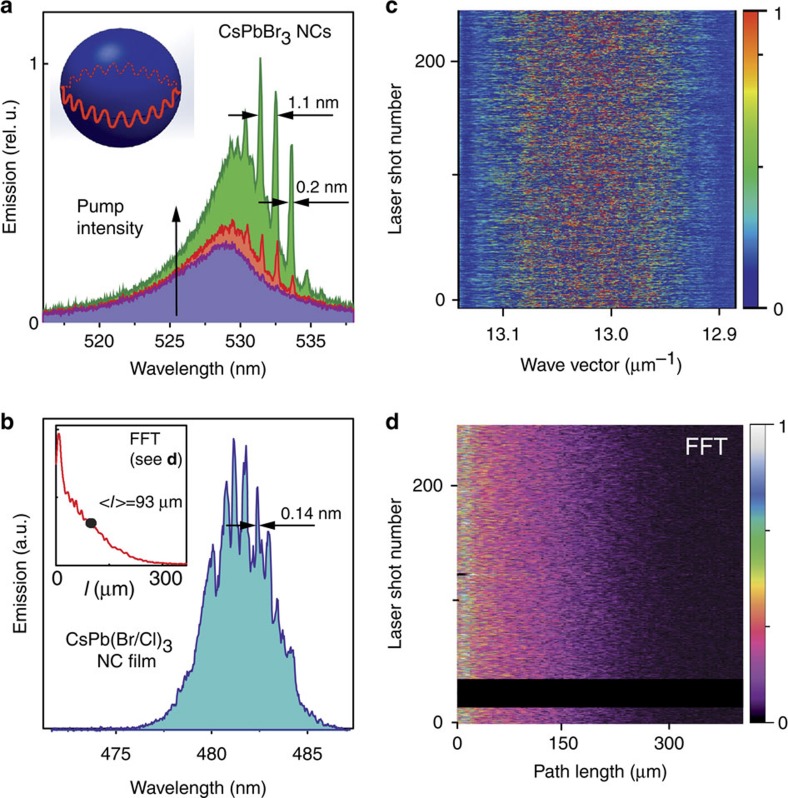
Lasing in perovskite CsPbX_3_ NC films. (**a**) Evolution from PL to whispering-gallery-mode (WGM) lasing with increasing pump intensity in a microsphere resonator of 15 μm in diameter, covered by a film of CsPbBr_3_ NCs. (**b**) Single pump laser shot mode structure of random lasing from CsPb(Br/Cl)_3_ NC film. The inset shows path-length distribution averaged over 256 pump laser shots. (**c**) Stochastic mode distribution in a series of 256 pump laser shots (PL background emission is subtracted). (**d**) Fast Fourier transform (FFT) of (**c**).

Effective optical feedback from a high-quality optical resonator is needed to obtain lasing. In this regard, commercially available silica microspheres can serve as circular cavities in which the emitted light orbits around the circumference due to total internal reflection (Fig. 5a, inset). The resulting cavity modes are known as WGMs. WGM lasers can be conveniently observed by the adhesion of solution-processed lasing material onto the surface of the microspheres^17^,^40^,^41^. The great utility of microsphere resonators for research purposes stems from their extremely high and wavelength-independent *Q* factors of up to 10^9^ (describing the degree of feedback of the cavity), and their rather isotropic leakage of the emitted light. In this work, we obtained well-resolved lasing modes with pumping threshold behaviour using 15 μm (Fig. 5a) and 53 μm (Supplementary Fig. 9) spheres, with intermodal distances dependent on the sphere diameter (the larger the sphere, the smaller the spacing). All spectra were excited at the wavelength 400nm with 100fs laser pulses. The observed linewidths of lasing modes (0.15–0.20 nm) are limited primarily by the resolution of the spectrometer used for detection. We note that the spectra presented herein were collected from single microspheres using a microscope objective. In contrast, when the emission from several spheres was integrated, lasing modes were often indistinguishable due to the small but essential standard size-deviation of the spheres of *ca.* 0.5–1%.

### Random lasing from CsPbX_3_ nanocrystals

Lasing can also be observed without optical resonators, namely when the required optical feedback is provided via light scattering induced by intrinsic disorder in the lasing medium, leading to so-called random lasing^42^. Light diffuses in highly scattering media and randomly forms closed loops causing random fluctuations of lasing modes. Scattering occurs, for instance, on the aggregates of NCs, and is clearly pronounced in thicker films of several microns. Since the path of the light is unique and irreproducible, so are the lasing modes (Fig. 5b) generated by each shot of the pumping laser. For the CsPbX_3_ NC films investigated in this work, the modes appear to be fully stochastic and their distribution for 256 consecutive laser shots is presented in Fig. 5c.

The multiple emission spectra expressed as a function of wave vector in k space, shown in Fig. 5c, can be Fourier transformed into a corresponding optical path-length distribution (Fig. 5d). The averaged distribution of path length, *l*, over 256 shots is presented in the inset of Fig. 5b. The mean path length, *<l>*=93±5 μm, is obtained by integral averaging over the path-length range of 0–300 μm. The criterion for Anderson localization, *k*<*l*>≈10^3^, is much larger than unity, pointing to the case of weakly scattering random lasing. The medium is rather transparent and shows no apparent effects from film imperfections such as cracks and pinholes. The lasing threshold is of the same order of magnitude as ASE threshold discussed above. Due to the strong increase in intensity of Rayleigh scattering with decreasing wavelength (*I*∝*λ*^−4^), aggregation-induced scattering and hence random lasing is most pronounced in blue-emitting samples, such as the one shown in Fig. 5b.

## Discussion

Considering the colloidal nature of CsPbX_3_ NCs, the most relevant comparison to be drawn is with strongly quantum-confined Cd-chalcogenide-based colloidal NCs. After the first demonstration of optical gain and stimulated emission from colloidal CdSe NCs (emission at 620 nm) in 2000 (ref. 37), colloidal NCs have been considered as an eventual alternative to more expensive epitaxial group III–V materials (for example, InGaN, GaAsP and InGaAs). The green spectral region is especially difficult to access by III–V compounds^43^. For this reason, the size-tunable emission of Cd-chalcogenide-based materials is highly appealing, but still lacks stability in the blue spectral region (≤500 nm)^29^. So far, the lowest pumping thresholds of Cd-based quantum dots (QDs) have been reported for specially engineered CdSe/ZnCdS core-shell structures, ranging from 90 μJ cm^−2^ for red QDs to 800 μJ cm^−2^ for blue QDs with single-exciton nature of the optical gain^28^. Recently, a large step forward was made by introducing pristine CdSe and core-shell CdSe/CdS nanoplatelets^35^,^44^,^45^, showing the lowest ASE thresholds for inorganic colloidal nanomaterials obtained to date (6 μJ cm^−2^ at 520 nm and 8 μJ cm^−2^ at 635 nm)^34^,^35^. Furthermore, in such atomically flat CdSe nanoplatelets, where bandgaps are tunable stepwise by adjusting the number of unit cells in platelet thickness, only discrete emission wavelengths were so far demonstrated and ASE had not been reported below 510 nm. Overall, in Cd-chalcogenide-based systems, emission wavelength tuning is almost exclusively achieved via quantum-size effects. On the contrary, in this study rather large CsPbX_3_ NCs (9–10 nm) with weak to no quantum confinement were chosen and the emission was found to be freely adjustable via compositional tuning (that is, by the halide ratio). Such convenient compositional tuning is not easily accessible in Cd-chalcogenide NCs. In experiments on smaller CsPbBr_3_ NCs with pronounced quantum-size effects, by up to an order of magnitude higher ASE thresholds were observed. Thus, a clear complementarity is seen between weakly confined CsPbX_3_ NCs (performing best in the blue–green with ≤10 μJ cm^−2^ blue ASE thresholds) and strongly confined Cd-chalcogenide-based materials (performing best in the green–red with ≤10 μJ cm^−2^ ASE thresholds for red CdSe/CdS platelets). For a completely unbiased comparison, we have reproduced the synthesis of CdSe and core-shell CdSe/CdS nanoplatelets described by She *et* *al.*^35^, and observed same low thresholds of 7–15 μJ cm^−2^ (Supplementary Figs 10 and 11) under the same testing conditions as applied here for CsPbX_3_ NCs using femtosecond excitations. For nanosecond excitation pulses (Fig. 4), ASE thresholds for CsPbX_3_ NCs rise to ∼0.45 mJ cm^−2^ (and 1 mJ cm^−2^ for CdSe/CdS nanoplatelets; see Supplementary Fig. 12). Low ASE thresholds in CsPbX_3_ NCs are also assisted by an extremely large absorption cross-section (σ). For CsPbBr_3_ perovskite NCs, we estimated *σ*= 8 × 10^−14^ cm^2^ (or 8 nm^2^), that is almost two orders of magnitude larger than typically reported for CdSe QDs^46^, and similar to CdSe nanoplatelets^47^, emitting in the same wavelength range (green). No continuous-wave ASE could be observed at room temperature or down to 80 K for neither CsPbX_3_ NCs nor for Cd-chalcogenide platelets, up to excitation levels causing photo-damage of the samples.

A comparison of our results for CsPbX_3_ NCs with reports on closely related solution-deposited hybrid perovskite MAPbX_3_ films and microcrystals points to rather similar ASE thresholds (Supplementary Table 1)^17^,^18^,^19^,^20^. MAPbX_3_ films were also reported to exhibit random lasing^48^, lasing from vertical cavities^20^ and from spherical resonators^17^ and WGM lasing^19^. The comparably good performance of CsPbBr_3_ NCs indicates that their high surface area does not impede their optical properties. Also the solution-processed organic semiconductor materials exhibit similar ASE thresholds under similar testing conditions (no cavity, plane wave-guiding films)^49^. So far, in only a few examples of vacuum-deposited organic semiconductors^50^ and epitaxial multiple-quantum-well structures^51^ were lower, sub-μJ-cm^−2^ ASE thresholds demonstrated, but usually with an order of magnitude lower optical gain values. At the time of the submission of this work, much lower thresholds (0.22 μJ cm^−2^) were reported by Zhu *et al.*^21^, for lasing from MAPbI_3_ wires, highlighting also the morphological effects (wire acts as a single-mode waveguide and laser resonator) and suggesting that future studies should focus on engineering CsPbX_3_ wire-like morphologies with dimensions comparable to the wavelengths of light. As a first step in this direction and due to the lack of reports on bulk-like CsPbX_3_, we have prepared CsPbX_3_ polycrystalline films (with crystalline domain sizes of several μm) and individual microcrystals simply by drop-casting from dimethylformamide solutions and inspected their PL/ASE characteristics. NCs and microcrystals of the same composition exhibit nearly identical PL peak wavelengths and linewidths, and radiative lifetimes (Supplementary Fig. 13). ASE thresholds of polycrystalline films were by an order of magnitude higher, but this may well be caused also by the difficult-to-control, suboptimal for ASE build-up morphologies of these films. Similarly to CsPbX_3_ NCs, the ASE peaks were found to be red-shifted with respect to the PL maxima (Supplementary Fig. 14). For selected large microcrystals (100–150 μm) clear WGM lasing modes were observed as well (Supplementary Fig. 15). No wire-like morphologies could be found for direct comparison with MAPbI_3_ wires. Overall, we conclude that among the three distinct cases—small CsPbX_3_ QDs (4–8 nm, chemically unstable), CsPbX_3_ NCs (10 nm) and CsPbX_3_ microcrystals—the NCs exhibit the best balance between optical performance (having generally the lowest ASE thresholds) and chemical versatility (exhibiting facile solution processing, and easily adjustable film thickness and morphology for obtaining various lasing regimes).

At present, we cannot fully answer the remaining question of this study—the exact mechanism for optical gain. This is also an open question in the MAPbX_3_-related literature. To speculate on this matter for CsPbX_3_ NCs, the observed red shift of the ASE peaks with respect to PL maxima can be explained by ether bi-excitonic lasing (due to binding energy of bi-exciton)^37^ or by self-absorption in the case of a single-exciton gain^28^,^29^. We also evaluated the average density of excitons per each NC (*<N>*, for details, see Supplementary Note 1) at the ASE threshold and found *<N>*=0.5±0.15. Expected theoretical values of *<N>* are 0.5 for single-exciton gain^29^ and 1 for bi-excitonic mechanism^37^,^52^. Increase of QY with pump intensity (Supplementary Fig. 16) might be another plausible evidence for single-exciton gain, as QY should decrease when Auger recombination is limiting the ASE (typically observed for bi-excitonic gain).

In summary, perovskite CsPbX_3_ NCs, synthesized via a simple one-step reaction between PbX_2_ and Cs-oleate in nonpolar solvent media, are particularly promising for achieving ASE/lasing in blue and green spectral regions. Optical gain is demonstrated here at room temperature for pulse durations of up to 10 ns, corresponding to the quasi-continuous wave (quasi-cw) excitation regime with low ASE threshold values down to 5 μJ cm^−2^. High optical gain values of up to 450 cm^−1^ allow for obtaining resonant conditions for lasing either by coating CsPbX_3_ NCs onto spherical microresonators or via random lasing mediated by light scattering on NC aggregates. Random lasing, arising from the combined effect of wave guiding and light scattering in the optical gain medium of densely packed aggregates of CsPbX_3_ NCs, does not require an ultraprecise cavity as in conventional lasing. This not only provides the obvious technological advantage of facile and inexpensive fabrication, but also enables various niche applications such as displays and lighting, benefitting from the broad spectral angular distribution of the random lasing^42^,^53^. Another promising area of application for random lasers, benefitting from the broad ASE spectrum, is as the light source in optical coherence tomography where it is critical to keep optical coherence moderately low but controlled^54^,^55^. Random lasing in the weak-scattering regime, where the spectral distribution of lasing modes is unique for each laser shot, can serve as a physically based method for random number generation and in cryptography^56^.

## Methods

### Preparation of CsPbX_3_ NCs and microcrystalline films

CsPbX_3_ NCs were synthesized as described in our recent report^22^. The crude solution was cooled down with water bath and aggregated NCs were separated by centrifuging for 3 min at 11,000 r.p.m. After centrifugation, the supernatant was discarded, the particles were redispersed in 0.3 ml hexane and centrifuged again for 4 min at 12,000 r.p.m. After repeating the previous step one more time, the precipitate was redispersed in 0.6 ml toluene, and 0.2 ml acetonitrile was added for precipitation. The NCs were centrifuged again for 4 min at 12,000 r.p.m., and after this, the supernatant was discarded and the precipitate was redispersed in toluene. Thin films of CsPbX_3_ NCs were obtained by drop-casting 10–50 μl of CsPbX_3_ NC solution at ambient conditions onto glass substrates, followed by drying at ambient conditions. For coating CsPbX_3_ NCs onto silica microspheres, water-dispersed 15- and 53-μm silica microspheres (http://www.microspheres-nanospheres.com) were first drop-cast onto a hot glass substrate, followed by drop-casting of CsPbX_3_ NCs. For preparing microcrystalline films, PbX_2_ and CsX were dissolved in dimethylformamide, and then drop- or spin-cast onto a glass substrate followed by heat treatment at 150 °C. Alternatively, films of PbX_2_ can be converted into CsPbX_3_ on dipping into Cs-halide solutions.

### Characterization of CsPbX_3_ NC solutions and films

UV-Vis absorption and reflection spectra of the NC films were collected using a Jasco V670 spectrometer equipped with an integrating sphere. Steady-state PL emission and excitation spectra were acquired with a Fluorolog iHR320 Horiba Jobin Yvon spectrofluorometer, equipped with Xe lamp and a photomultiplier tube (PMT) detector. PL lifetime measurements were performed using a time-correlated single-photon counting setup, equipped with SPC-130-EM counting module (Becker & Hickl GmbH) and an IDQ-ID-100-20-ULN avalanche photodiode (Quantique) for recording the decay traces. The emission of the perovskite NCs was excited by a 400-nm 100-fs laser pulses with a repetition of 1 kHz synchronized to time-correlated single-photon counting module through an electronic delay generator (DG535 from Stanford Research Systems). Transmission electron microscopy images were recorded using a JEOL JEM-2200FS microscope operated at 200 kV. For the thickness determination of the films, an AlphaStep D-120 profilometer was used.

### ASE and lasing experiments

These experiments were performed with excitation light from nanosecond and femtosecond lasers. All experiments were conducted at room temperature. The femtosecond laser system consisted of an oscillator (Vitesse 800) and an amplifier (Legend Elite), both from Coherent Inc., with a frequency-doubling external beta barium borate (BBO) crystal; it yielded 100 fs pulses at 400 nm, with a repetition rate of 1 kHz and pulse energy of up to 4 μJ. The laser beam profile had a TEM_00_ mode with a 1-mm FWHM diameter. Laser power was measured by a LabMax-TOP laser energy meter (Coherent Inc.) with a nJ-measuring head. For variable stripe length (VSL) experiments, the beam was focused into a stripe by a cylindrical lens with a focal length of 75 mm. The nanosecond laser system was a Quanta-Ray Pro 230-50 (Spectra-Physics), frequency tripled to 355 nm, with a pulse duration of 10 ns and a top-hat beam profile focused to a spot of 1.5 mm in diameter with a pulse energy of up to 30 μJ. To resolve the spectrally sharp lasing peaks, the optical emission was coupled into a Princeton Instruments SP-2300i spectrometer, equipped with a Thorlabs LC100/M CCD detector array (0.14 nm resolution). Fast Fourier Transform (FFT) analysis of random lasing mode patterns for large arrays of pump laser shots was performed using the Gwyddion software package. The laser beam intensity profiles were analysed by a LabMax-TOP camera from Coherent Inc.

## Additional information

**How to cite this article:** Yakunin, S. *et al.* Low-threshold amplified spontaneous emission and lasing from colloidal nanocrystals of caesium lead halide perovskites. *Nat. Commun.* 6:8056 doi: 10.1038/ncomms9056 (2015).

## Supplementary Material

Supplementary Information Supplementary Figures 1-16, Supplementary Table 1, Supplementary Note 1 and Supplementary References.
