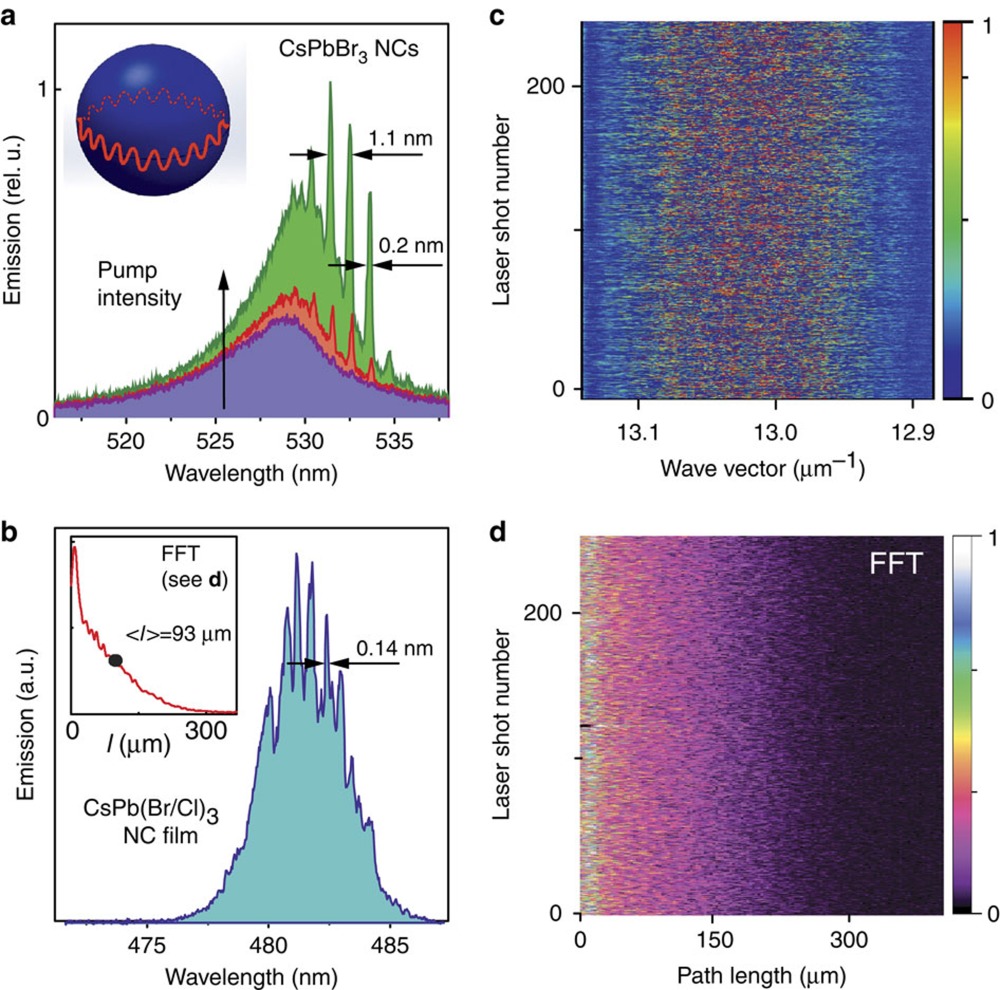# Erratum: Low-threshold amplified spontaneous emission and lasing from colloidal nanocrystals of caesium lead halide perovskites

**DOI:** 10.1038/ncomms9515

**Published:** 2015-09-23

**Authors:** Sergii Yakunin, Loredana Protesescu, Franziska Krieg, Maryna I. Bodnarchuk, Georgian Nedelcu, Markus Humer, Gabriele De Luca, Manfred Fiebig, Wolfgang Heiss, Maksym V. Kovalenko

*Nature Communications* 6: Article number: 805610.1038/ncomms9056 (2015); Published: 08202015; Updated: 09232015.

In Fig. 5d of this Article, a shaded box was inadvertently placed over the lower part of the panel during the final stages of production. The correct version of the figure appears below.

**Figure 5**