# Supplementary material for: Low-threshold amplified spontaneous emission and lasing from colloidal nanocrystals of caesium lead halide perovskites
Source: Nat Commun. 2015 Aug 20;6:8056. doi: 10.1038/ncomms9056 (PMC4560790; doi:10.1038/ncomms9056)
Supplement: Supplementary Information — Supplementary Figures 1-16, Supplementary Table 1, Supplementary Note 1 and Supplementary References. [file ncomms9056-s1.pdf]

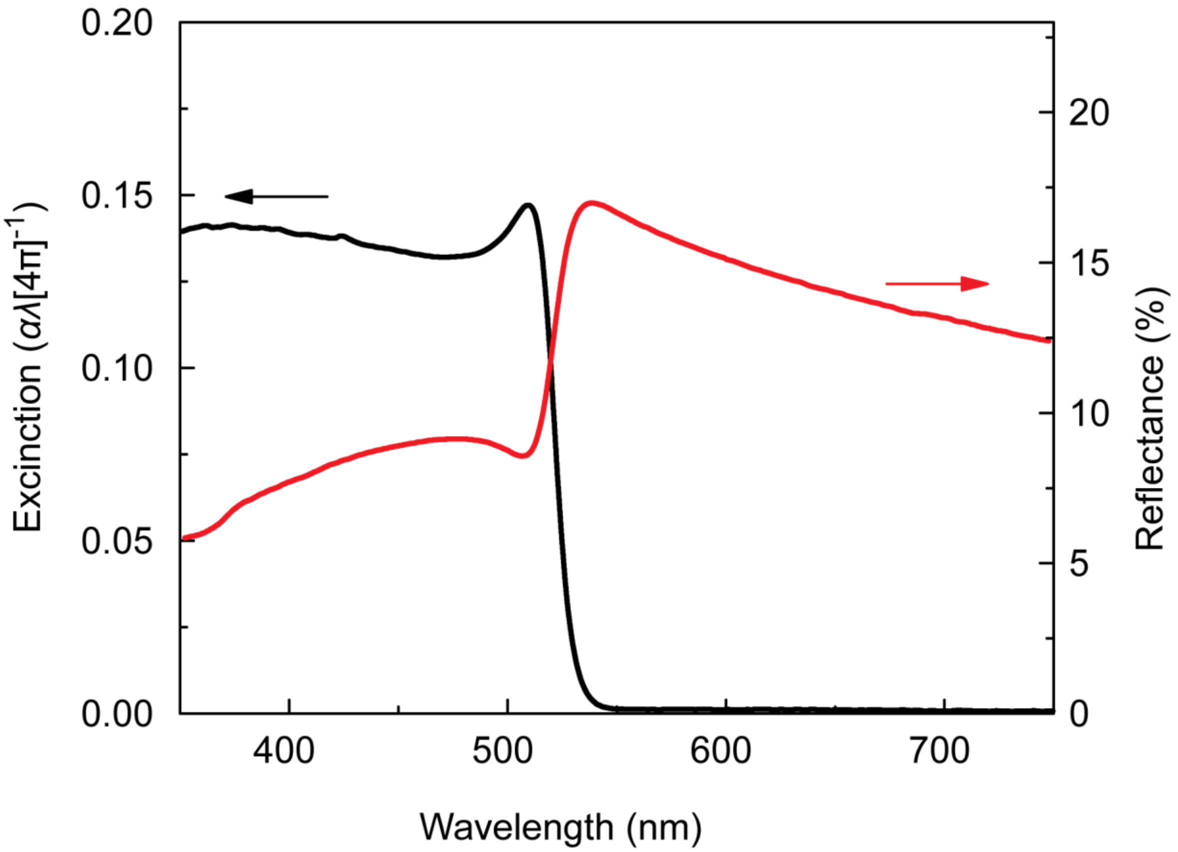

Supplementary Figure 1. Dispersion spectra of optical extinction (*i.e.*, the imaginary part of the complex refractive index, black line) and of reflectance for the perovskite $CsPbBr_3$ NC film. The refractive index was evaluated according to Supplementary Reference[1].

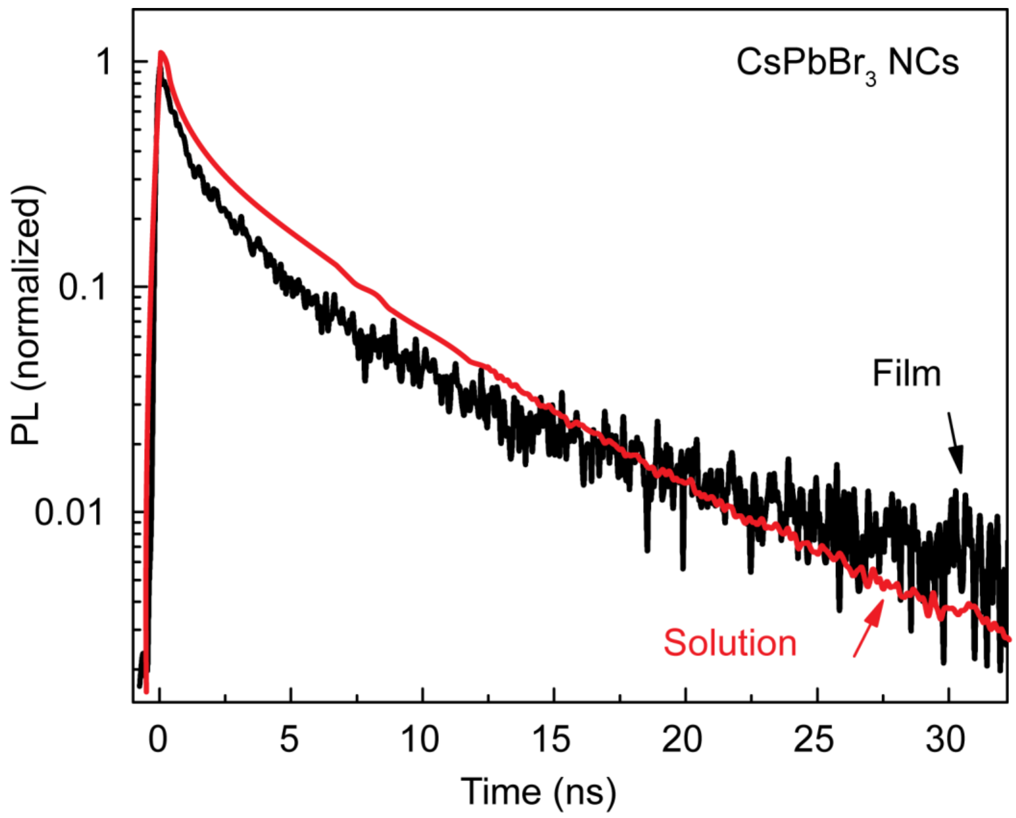

Supplementary Figure 2. Comparison of time-resolved photoluminescence decays for a $CsPbBr_3$ NC film (black line) and for a NC colloidal solution (red line). Excitation levels were well below the ASE threshold.

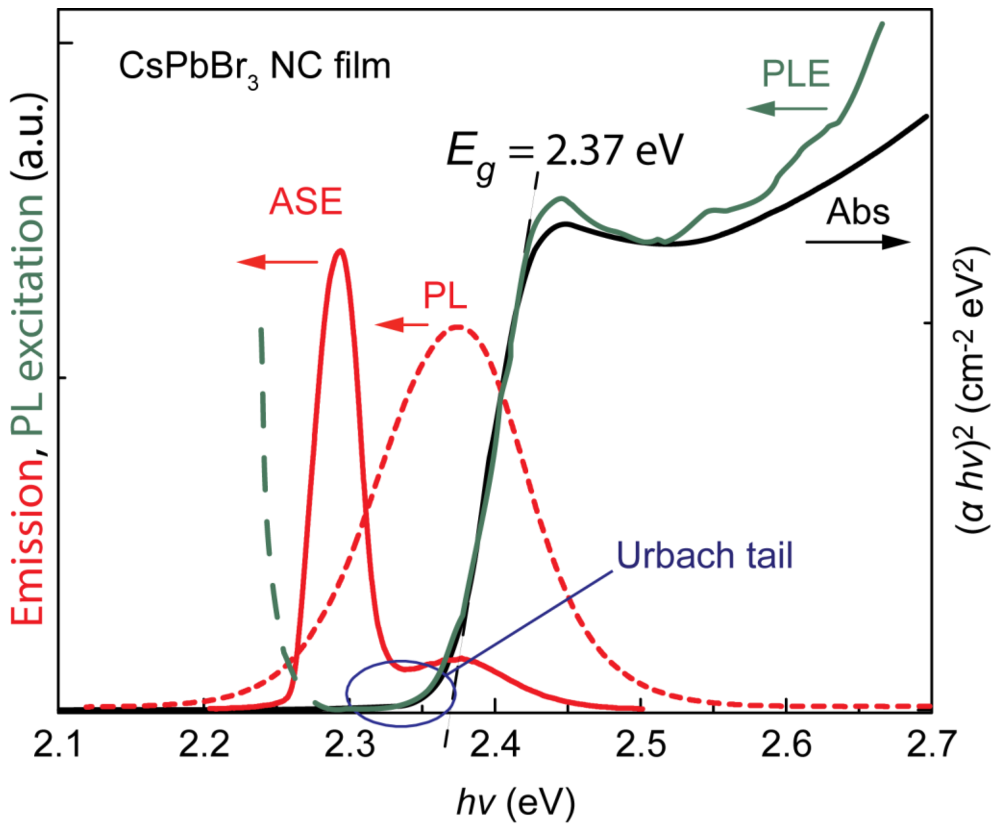

Supplementary Figure 3. For a $CsPbBr_3$ NC film, comparison of the ASE (solid red line), PL (dotted red line), PL excitation (solid green line, detection at 2.22 eV) absorption spectra (represented as a Tauc plot, black line).

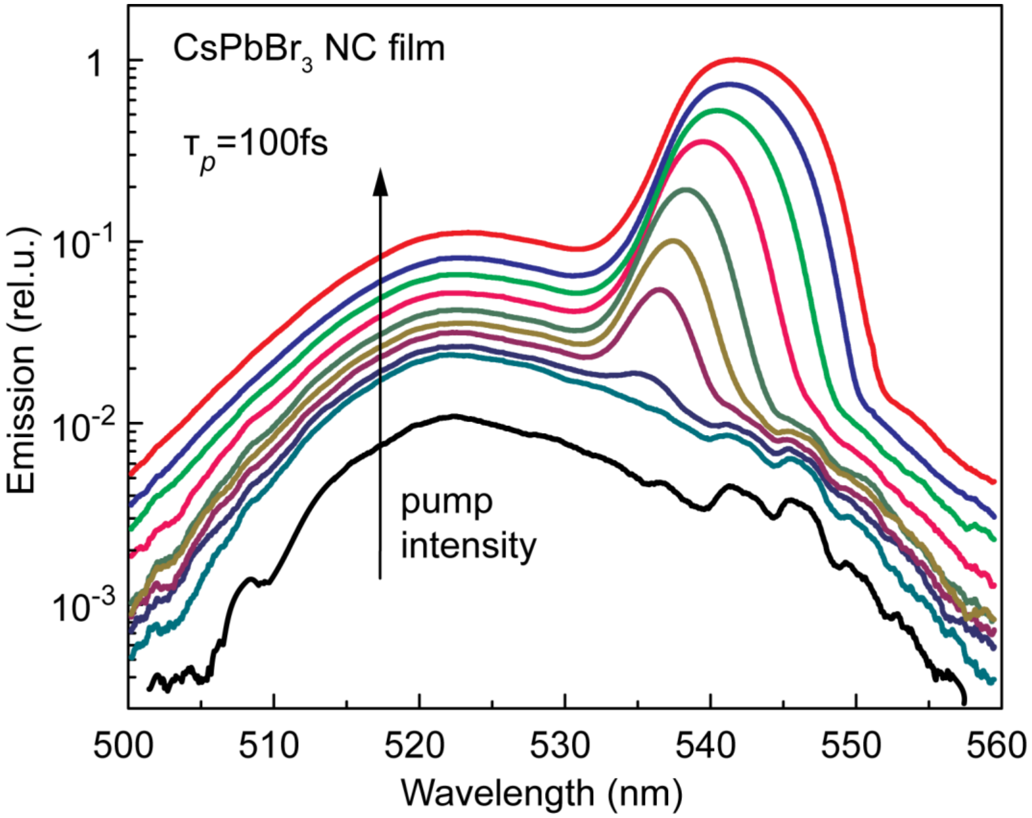

Supplementary Figure 4. Build-up of the ASE in the $CsPbBr_3$ NC film upon increase of pumping intensity from 3 up to 80 μJ cm^{-2}. The lowest five spectra correspond to Fig. 2a. At higher pump fluencies, the ASE spectrum broadens, indicating a greater fraction of the emission falling under optical gain conditions.

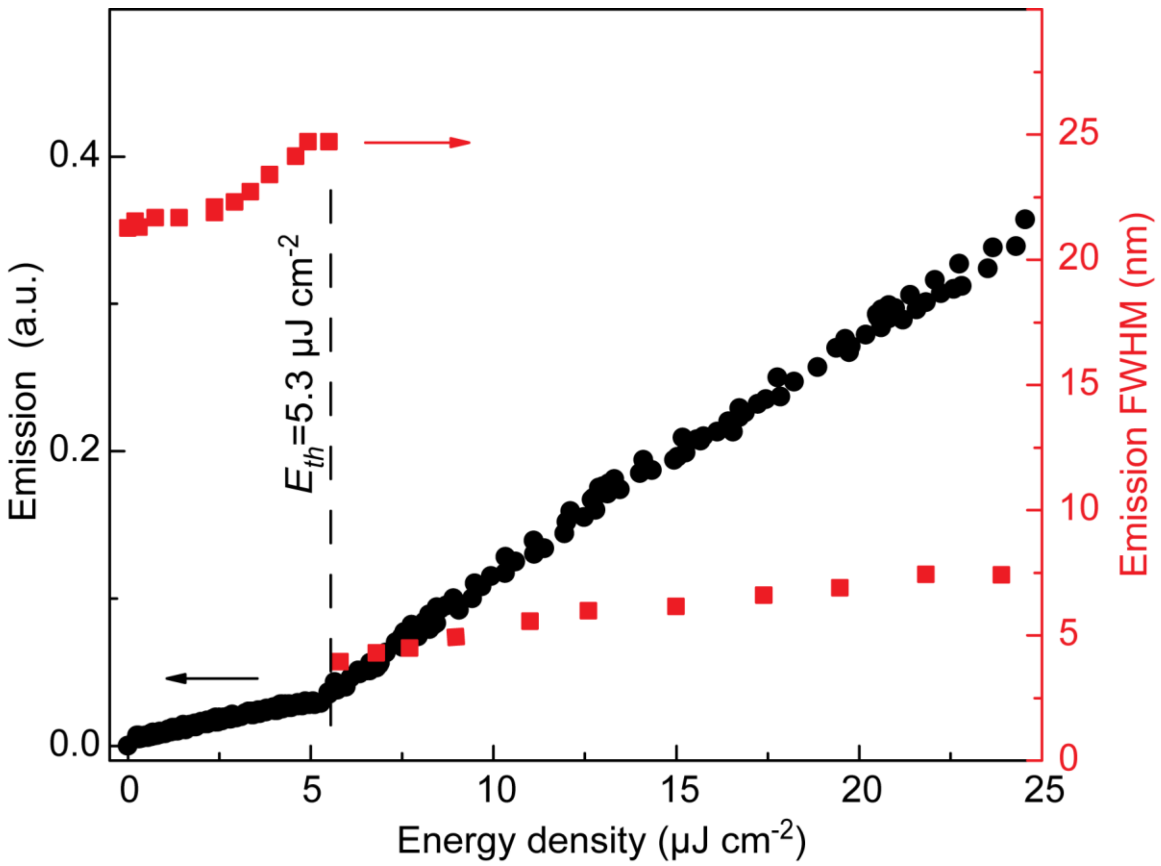

Supplementary Figure 5. Threshold behavior for intensity (black) and FWHM (red) of the PL/ASE emission for the $CsPbBr_3$ NC film (also presented in Fig. 2a, c).

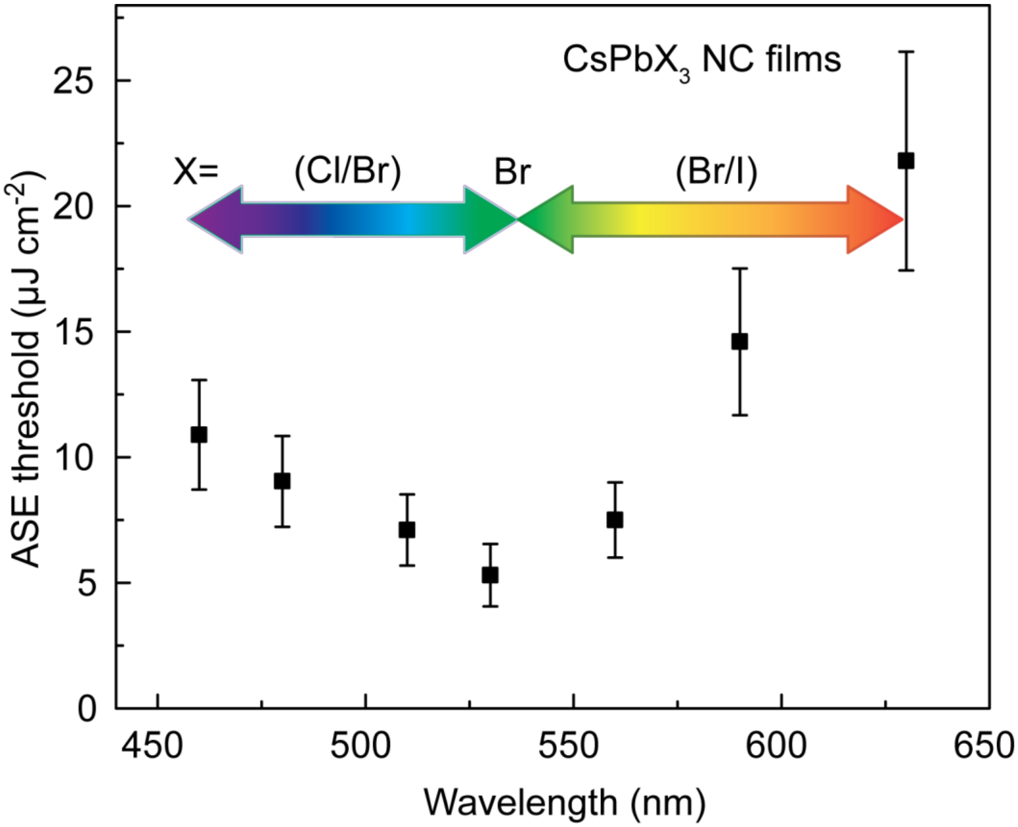

Supplementary Figure 6. ASE thresholds for $CsPbX_3$ NC films of different halide compositions. The lowest ASE thresholds were observed for the pure bromide system. Each data point is collected from 5-10 films.

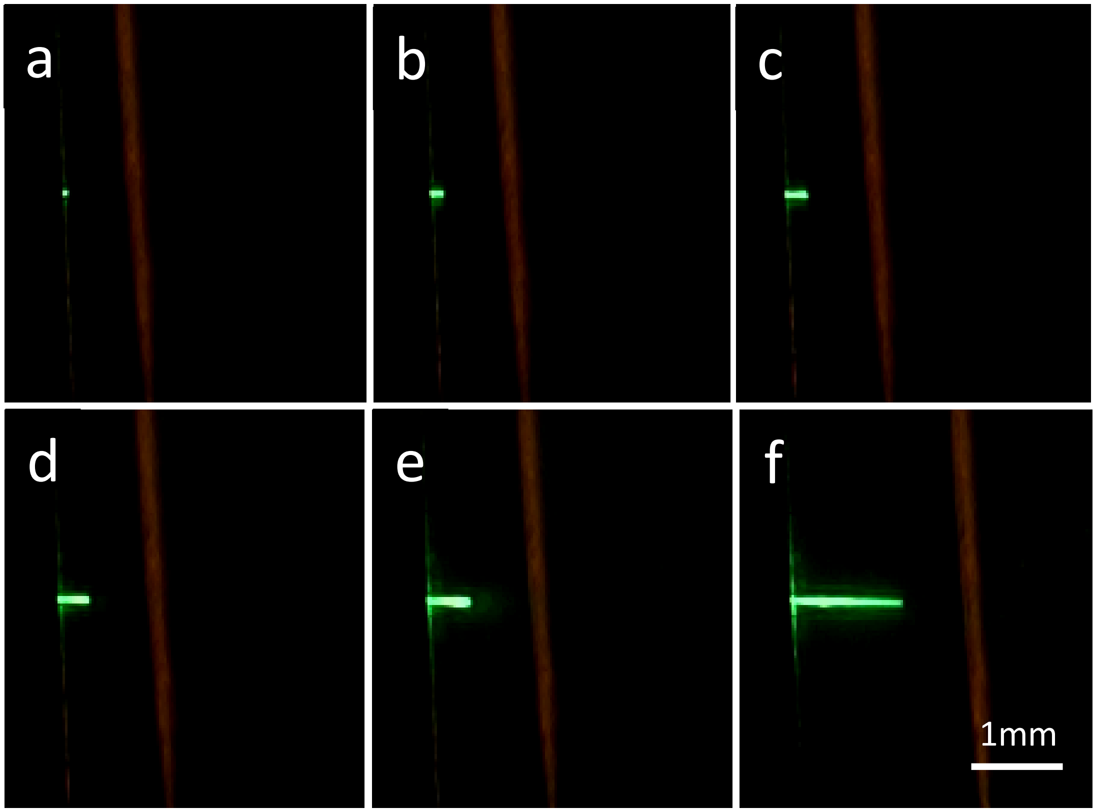

Supplementary Figure 7. Photograph of the shape of the emitting area in the variable stripe length (VSL) experiment. The excitation stripe demonstrates a rather uniform shape required for VSL experiments.

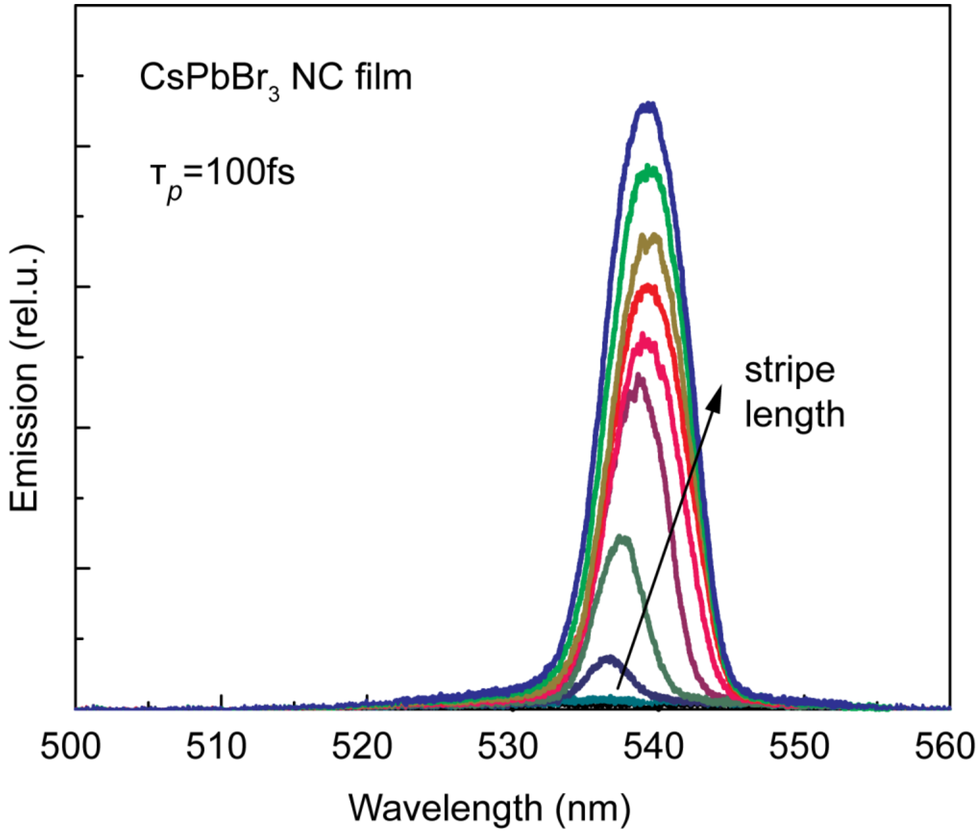

Supplementary Figure 8. Emission spectra in a VSL experiment with a $CsPbBr_3$ NC film. The spectra correspond to Fig. 2d and Supplementary Fig. 7. The red shift of the ASE band is due to wave-guiding losses in the $CsPbBr_3$ NC film.

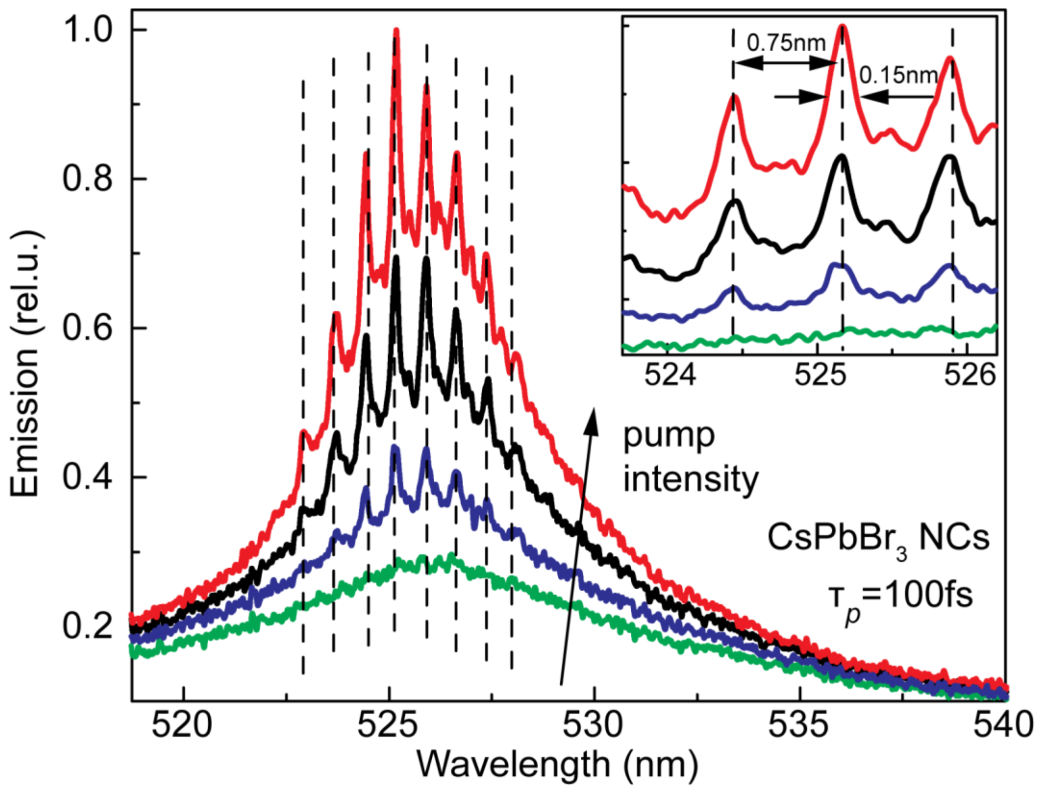

Supplementary Figure 9. Evolution of whispering gallery lasing modes with increasing pump intensity for microsphere resonator of 53 μm in diameter covered by $CsPbBr_3$ NCs. Inset shows zoom into the mode structure.

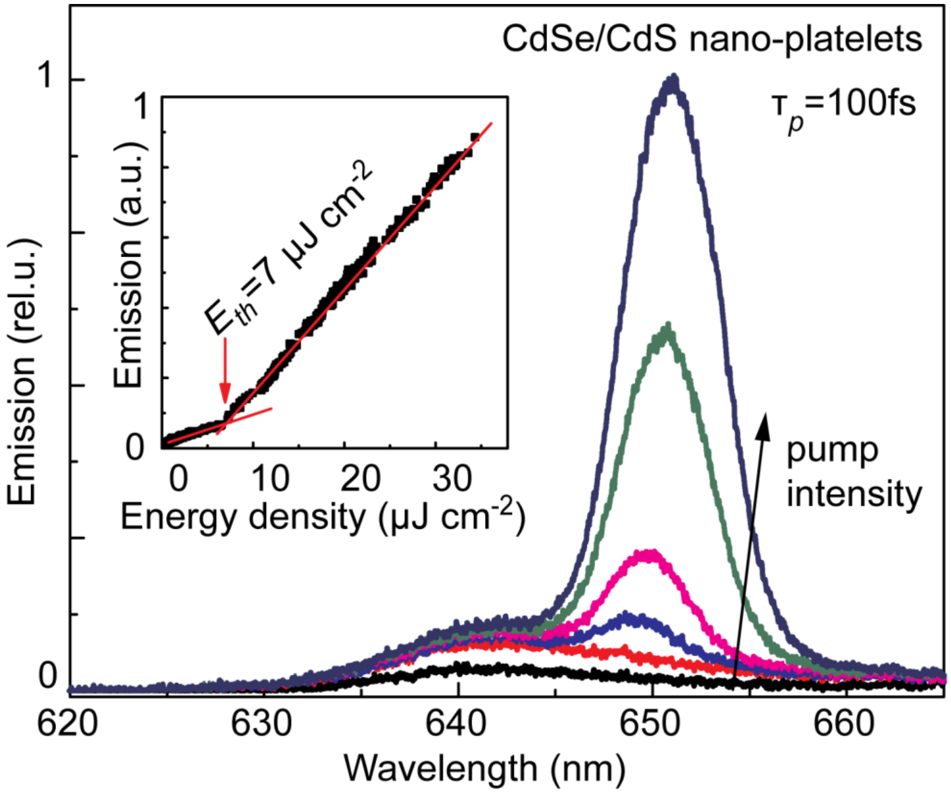

Supplementary Figure 10. PL/ASE spectra from CdSe/CdS core-shell nanoplatelets under fs laser pulses (λ=400 nm). The inset shows the ASE threshold. The ASE threshold values are similar to the 6 μJ cm^{-2} value reported in Supplementary Reference[2].

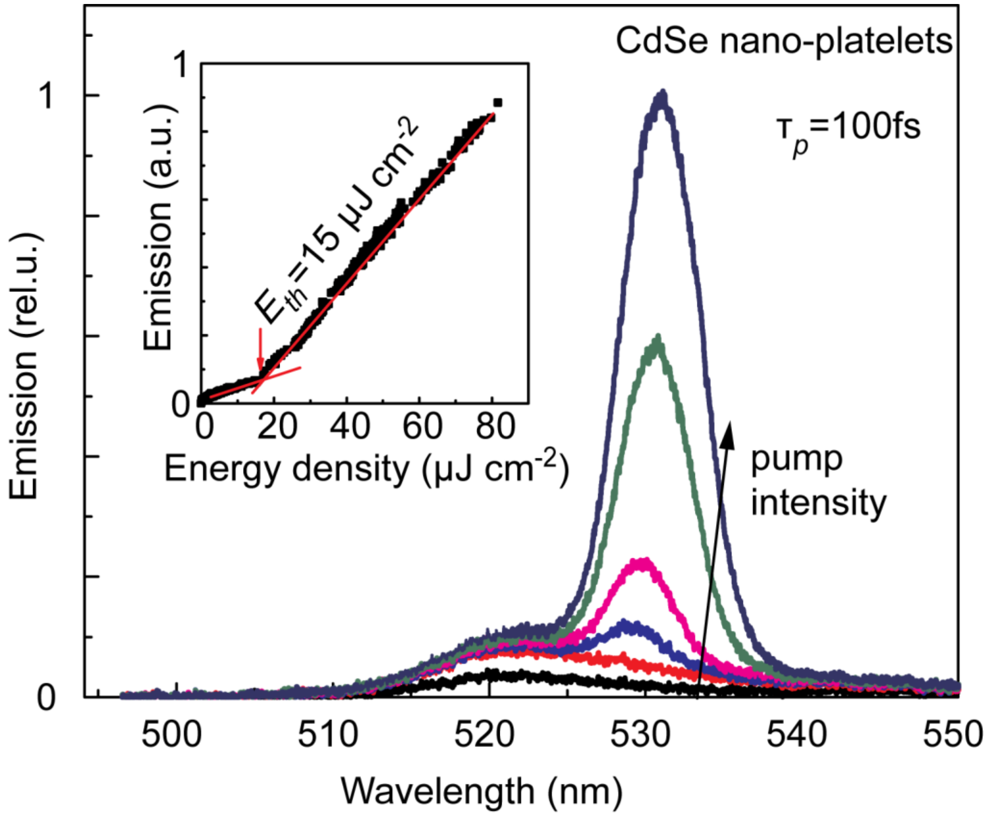

Supplementary Figure 11. PL/ASE spectra from CdSe core nanoplatelets under fs laser pulses (λ=400 nm). The inset shows the ASE threshold.

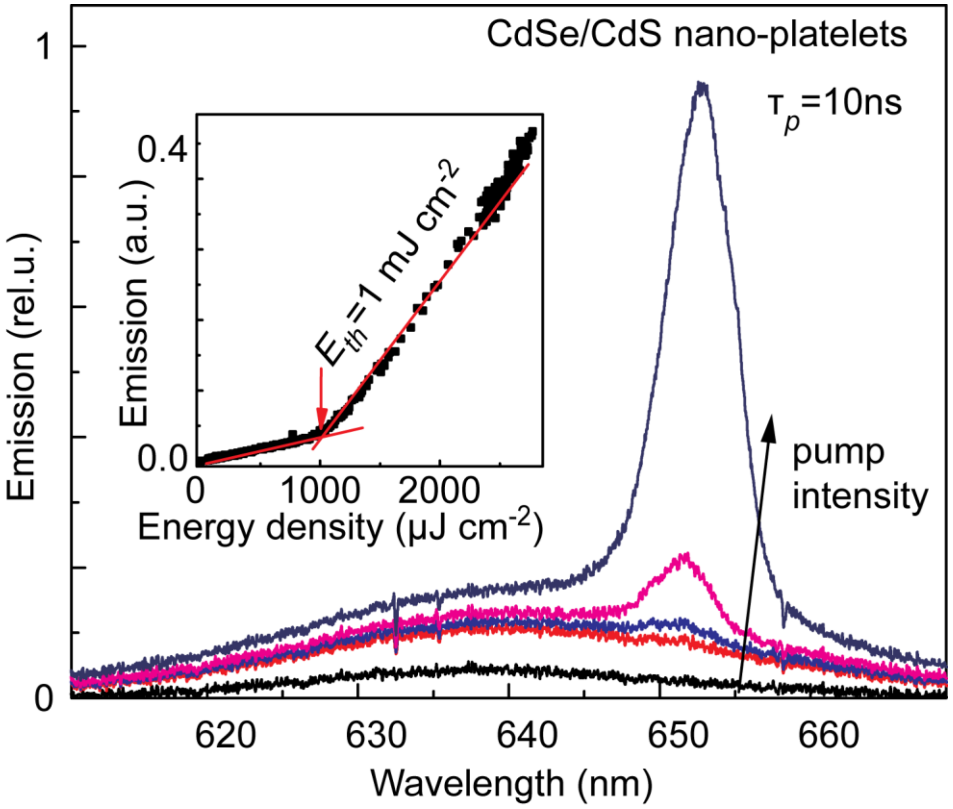

Supplementary Figure 12. PL/ASE spectra from CdSe/CdS core-shell nanoplatelets under ns laser pulses (λ=355 nm). The inset shows the ASE threshold.

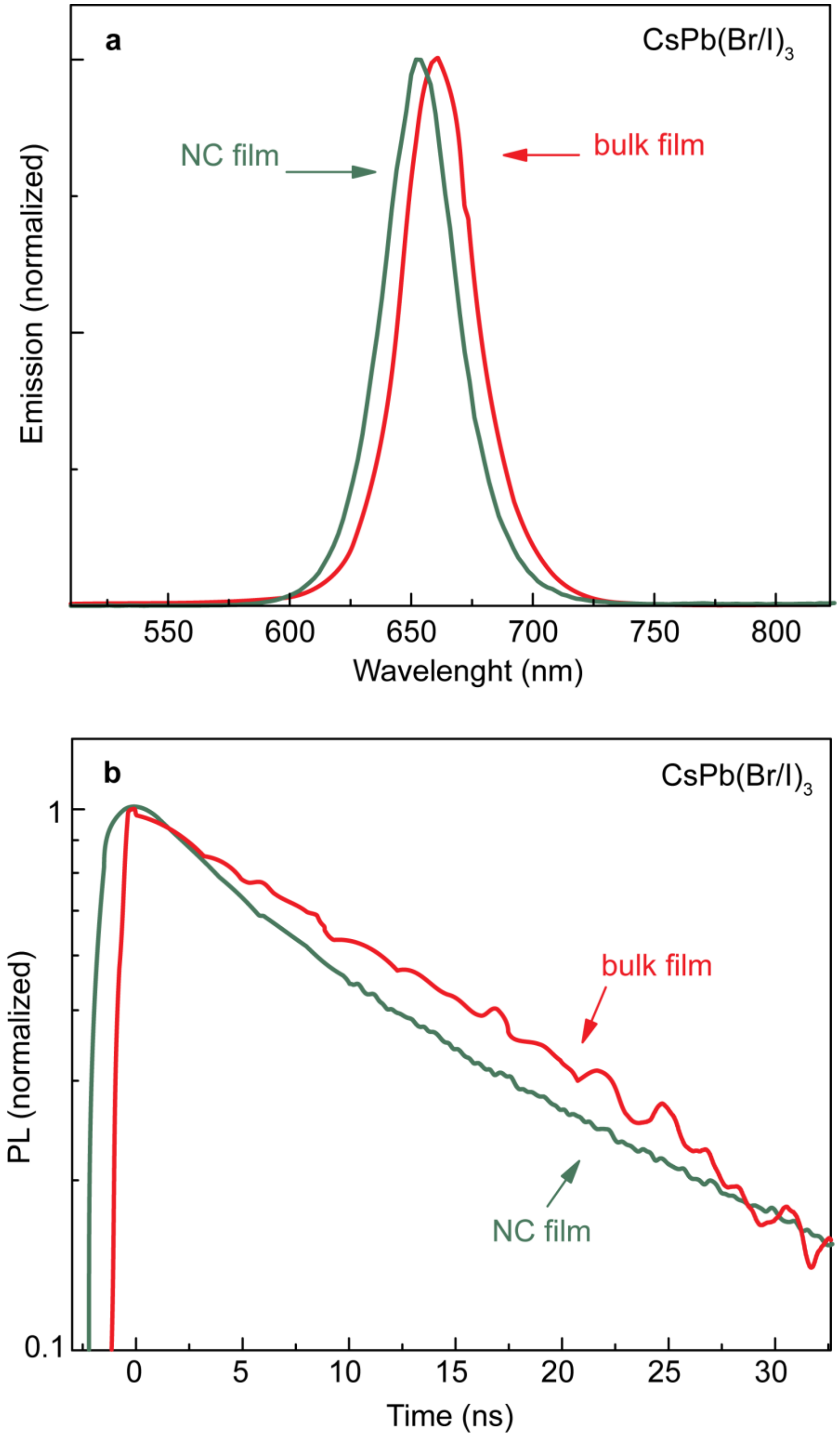

Supplementary Figure 13. Comparison of (**a**) PL spectra and (**b**) time-resolved PL decays for the $CsPb(Br/I)_3$ NC film (green line) and microcrystalline film (red line), showing similar radiative lifetimes of 16-17 ns.

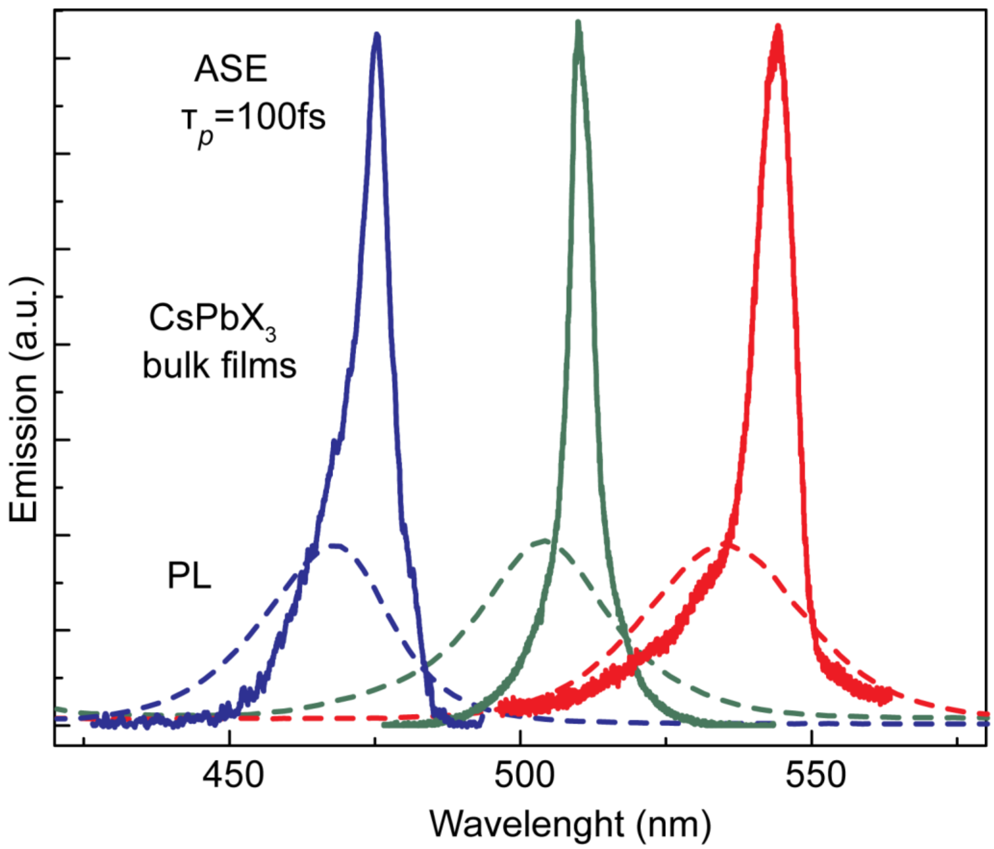

Supplementary Figure 14. Comparison of the ASE (solid lines) and PL (dashed lines) spectra for $CsPb(Cl/Br)_3$ of two compositions (blue and green curves; higher Cl content for shorter wavelength) and $CsPb(Br/I)_3$ (red curve) microcrystalline films.

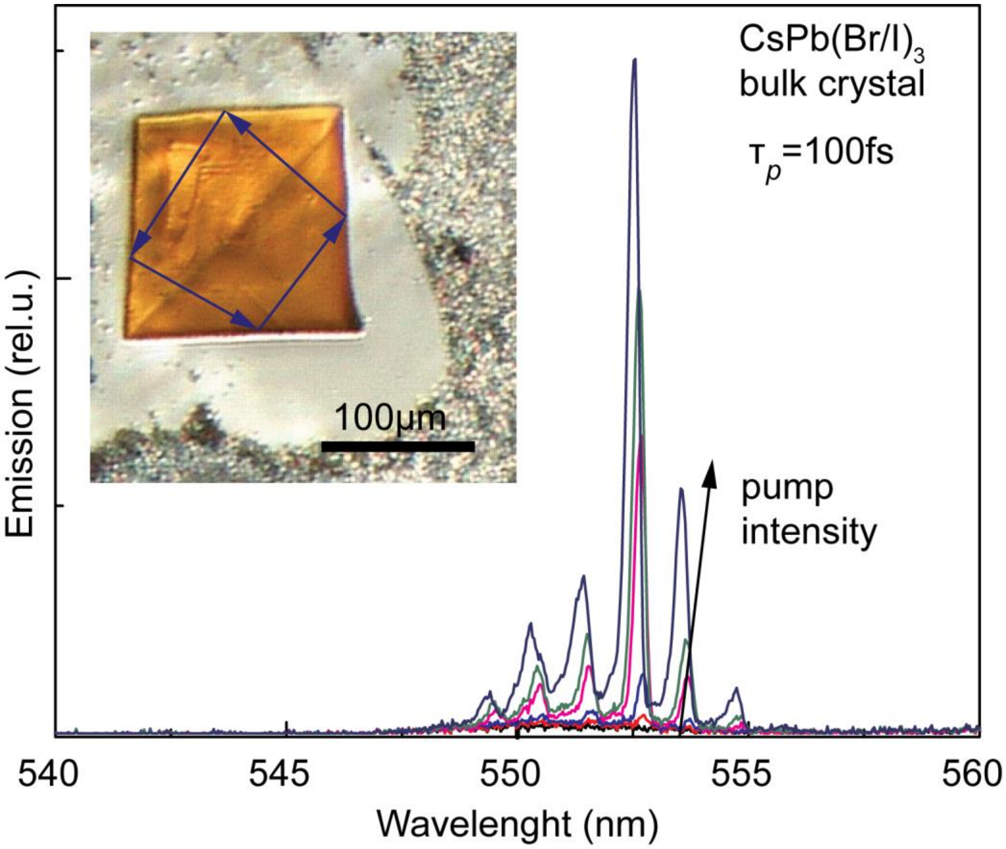

Supplementary Figure 15. Whispering gallery mode (WGM) lasing from a $CsPb(Br/I)_3$ perovskite microcrystal of *ca.* 150 μm in size under fs laser pulses (λ=400 nm). The inset shows a micrograph of the $CsPb(Br/I)_3$ perovskite microcrystal overlaid with a schematic of the closed path of the light inside the square-shaped resonator.

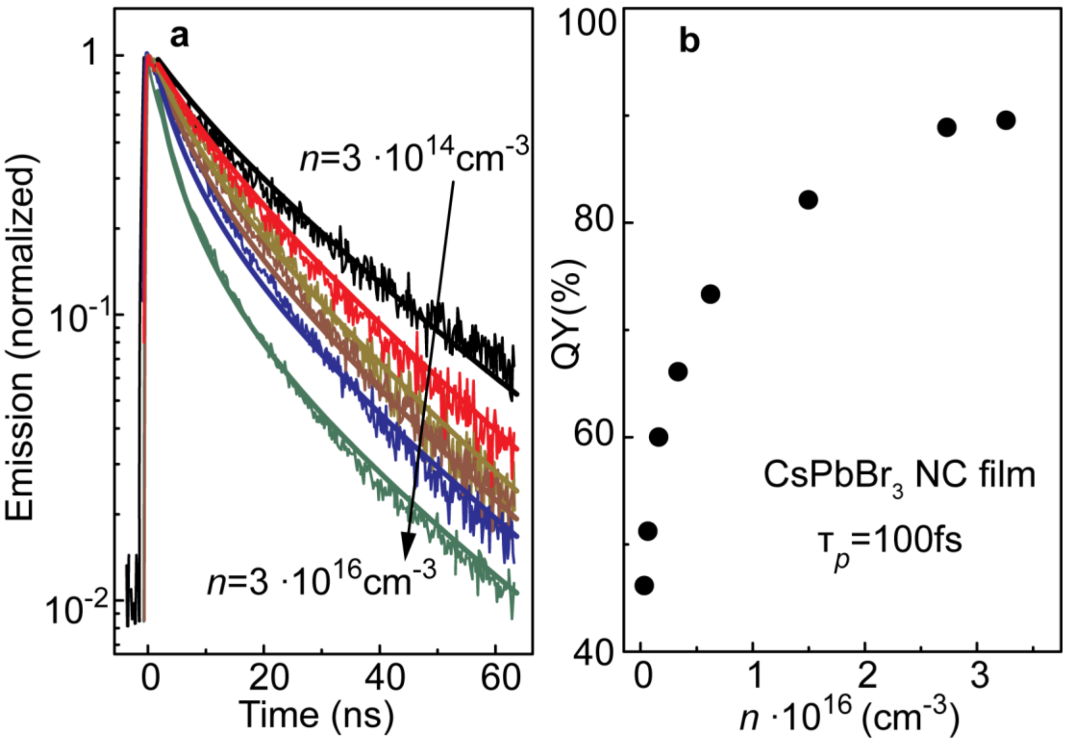

Supplementary Figure 16. (**a**) Time-resolved PL decays for the $CsPbBr_3$ NC film at increasing excitation intensity (represented via excited carrier density *n*). Thick solid lines are fits using AB-model.[3] Based on these fits, QYs were estimated and plotted in (**b**). Excitation levels were below the ASE threshold. For comparison, QYs were also measured directly using integrating sphere, yielding a factor of 2-3 lower values, but with the same trend of increase of QYs with the pumping intensity.

Supplementary Table 1. ASE and lasing characteristics of $MAPbX_3$ materials (literature data) and $CsPbX_3$ NCs (this study).

**Material,** X= Cl, Br, I	**Threshold**	**Pulse duration**	**Q-factor**	**Modal gain**	**Supplementary References**
$MAPbI_3$, films	65±8 $\mu J \cdot cm^{-2}$, ASE	2 ns	10^3	125±22 cm^{-1}	4
$MAPbX_3$, films	12±2 $\mu J\ cm^{-2}$, ASE	50 fs	-	250 cm^{-1}	5
$MAPbI_3$, platelets	40±1 $\mu J\ cm^{-2}$, lasing	50 fs	900	-	6
$MAPbI_3$, films	4 $\mu J\ cm^{-2}$, lasing	68 ps	-	-	7
$MAPbI_3$, nanowires	0.22 $\mu J\ cm^{-2}$, lasing	150 fs	≈3600	-	3
$CsPbX_3$, NCs	5±1 $\mu J \cdot cm^{-2}$, ASE	100 fs	≈3500	450±50 cm^{-1}	this study
$CsPbX_3$ NCs, film	450±50$\mu J\ cm^{-2}$, ASE	10 ns			this study

Supplementary Note 1. Calculation of the average density of excitons per density of NCs (<*N*>). <*N*> has been calculated as the ratio between the number of absorbed photons to the total number of NCs using at the fluence equal to ASE threshold with fs-pulses (that is pump pulse duration being well below radiative relaxation at ASE threshold of 300 ps). Taking into account the cubic shape of NCs and the ligand shell thickness of 1.5 nm, the density of NCs of about 1.8×10^{13} cm^{-2} was estimated for the studied NC film (d~0.4 μm). The density of absorbed photons (per pulse) at λ_{exc}=400 nm at ASE threshold energy density E_{th} for the same film is given by the formula: $N_{photons} = (1 - e^{-\alpha d})E_{th}\frac{\lambda}{hc}$, where α is optical absorption coefficient at λ=400 nm. Average <*N*> is thus **0.5±0.15** and only at the surface is about 0.8, where it is calculated as a product of incident photon density per pulse (1 10^{13} cm^{-2}) and absorption cross-section of $CsPbBr_3$ NCs (σ= 8 10^{-14} cm^2).

Supplementary References:

1 Leupacher, W. & Penzkofer, A. Refractive-index measurement of absorbing condensed media. *Appl. Opt.* **23**, 1554 (1984).
2 She, C. *et al.* Low-threshold stimulated emission using colloidal quantum wells. *Nano Lett.* **14**, 2772-2777 (2014).
3 Zhu, H. *et al.* Lead halide perovskite nanowire lasers with low lasing thresholds and high quality factors. *Nat. Mater.* **14**, 636-642 (2015).
4 Sutherland, B. R., Hoogland, S., Adachi, M. M., Wong, C. T. & Sargent, E. H. Conformal organohalide perovskites enable lasing on spherical resonators. *ACS Nano* **8**, 10947-10952 (2014).
5 Xing, G. *et al.* Low-temperature solution-processed wavelength-tunable perovskites for lasing. *Nat. Mater.* **13**, 476-480 (2014).
6 Zhang, Q., Ha, S. T., Liu, X., Sum, T. C. & Xiong, Q. Room-temperature near-infrared high-Q perovskite whispering-gallery planar nanolasers. *Nano Lett.* **14**, 5995-6001 (2014).
7 Deschler, F. *et al.* High photoluminescence efficiency and optically pumped lasing in solution-processed mixed halide perovskite semiconductors. *J. Phys. Chem. Lett.* **5**, 1421-1426 (2014).
